# Indiscriminate Extraction of the Six Year Molars Is a Sin, and Should Be Condemned

**Published:** 1868-02

**Authors:** G. A. Mills

**Affiliations:** Brooklyn


### INDISCRIMINATE EXTRACTION OF THE SIX YEAR MOLARS IS A SIN, AND SHOULD BE CONDEMNED.

BY G. A. MILLS, BROOKLYN.

*My Dear Register:* In reading of care and treatment of six year molars in the January number, my spirit groaned within me, and I asked myself, how long? Yea, oh Lord, how long will it be necessary for any one to speak out against the *damnable* practice of such indiscriminate extraction of these important organs of the human body? It does seem that the more sacred the temple, the greater the desire to desecrate. "We are living *truly* in a grand and awful time," and never has there been an hour in the history of the human race, when it was more necessary to preach the *truth; yea, knowledge* (is) nothing but *truth.* Why is it that our noble profession *seems* to be chosen (of God), I sincerely believe, to stand out in the front ranks and denounce the wholesale fractionalization of the tenement of the *Holy Ghost?* Why is it that so few *dare* to attempt, to even approximate towards as solemn a *duty* as was ever laid upon man? To cry out, to denounce, is the imperative demand of the hour. I say the Dentist seems to be chosen for this reason. It is admitted by those claiming to be high in authority in the medical profession, that we have made more advancement in the last twenty-five years than they have in two hundred. Now if this be half true, our calling is not one of drudgery. We read that "God made man in his own image," (this may refer to his moral character or form,) but he is the architect of the human form, and could we see man as he left his Maker's hands, none could *improve.* What would the architect of our day think, to see the symmetry of his designs deformed by the intrusion of the same misguided genius? Seeing an *angle* where a well rounded *curve* should have been, (and would, but for the blundering of an assistant,) endeavoring to improve the removal of a misdirected line, should leave a gap? It is a matter of astonishment to all, oftenest to the Dentist, that so many of our co-workers of *good* common sense in other directions, when coming to the practice of *even* trying to save the six year molar, do not stop to try, but coolly slip the forceps upon it, and laugh to see the trophy of their *hellish surgery.* Now did they stop here, it would be of some comfort, would with a dashing pace by an *unholy ambition*, too often (I fear), to be called *just*, or teachers of what? (error,) they dash on with a recklessness of a *Gilpin*, and scatter their infernal seed; but, thank God! the virgin soil does not readily embrace it in these days of quickening warmth and sunshine. The day of error is fast waning, and the time is nigh at hand, that he with open eyes may read as he runs, (and take notice of this fact,) that he who does not *run*, *can not* read. Truth means progress, and it does not look back to old and mildewed reputations, nor coats of arms of "ye olden times," but like the zealous Paul, although in evil and error at first, seeing his false position, rushes out of the broad road of wicked deeds, into the narrow pathway of light, accepting the blessed task of duty, though assured of trials, but ever balanced by the knowledge, *i. e.* truth, that comes to every regenerated soul; he counts all this as nought, that he may attain unto the treasure of fulfilling the highest point of Christianity, *i. e.* "to be of service to his fellow-man." Can we from this read no lesson of profit to our calling? Does our duty differ from Paul's? To be sure, we do not learn that he expresses Dentistry. But, fellow Dentists, he was a man under a solemn obligation to his Creator, to walk in the light. There is no need of this very thing. It is an evidence of darkness around and about, and a willingness to remain in it, when men shut their eyes to such acts of wrong doing in the direction to which this article points. The truth of these statements come by comparison; the facts are acknowledged by many a repentant one, and they are heard to say in their *manliness*, they will go and sin no more in the wholesale extraction of the first permanent molars.

